# Report of a Case of Congenital Cyanosis, with Remarks

**Published:** 1895-04

**Authors:** H. W. McLauthlin

**Affiliations:** Denver, Colo.


### REPORT OF A CASE OF CONGENITAL CYANOSIS, WITH REMARKS.

H. W. McLAUTHLIN, M. D., DENVER, COLO.

Joseph H., aged five and one-half years, born in the United States, is a twin child whose mate died a few minutes after birth. Nothing of importance appears in the family history. The parents are living and in good health. This child was always puny and delicate, and from the first it was noticed that his nails were blue and curved. When five months old, he had entero-colitis, and during his first and second summers was subject to attacks of diarrhoea. When four years old, he had whooping cough severely. He has never had rheumatism. His mother thinks he began to talk and that he cut his teeth at the usual time, but he did not walk until two and one-half years old, and had weakness of the ankles for a considerable time thereafter. General cyanosis first began to be noticed when he was two years old, especially after exertion, crying, anger or any excitement. The cyanosis increased and began to be attended with attacks of pain in the region of the heart, which would occur not only during the day, but at night, causing him to wake screaming and with the heart beating violently. At first these attacks of pain occurred only from once a week to once a month, but they have progressively increased until at present they often occur daily, and he is never free from them longer than a week. They not only come on when playing or when excited, but also when perfectly quiet and when asleep. When awake, these attacks of pain often cause him to fall powerless and screaming, but he has never lost consciousness nor had convulsions nor twitching. During the attacks he gets very blue and breathes heavily and quickly, while the heart beats violently. The attack lasts from five to fifteen minutes and is apt to recur after an interval of several minutes. He then becomes very drowsy, falls asleep, and when he awakes is in his normal condition except, perhaps, complaining of some headache or nausea or both. He complains much of feeling cold, and his feet and hands are always cold to the touch. The appetite is generally poor and the bowels are always constipated. He plays but little and always guardedly, and has dyspnoea on the slightest exertion. He always has some cough and takes cold easily. He is drowsy and sleepy much of the time and especially after a meal. He is easily irritated and often peevish. He seems bright and learns quite readily. The skin is always dry. He has never had oedema or dropsy. The urine shows nothing abnormal.

### PHYSICAL EXAMINATION.

Emaciation considerable; face of a bluish color as well as the hands and feet; lips, tongue, interior of mouth, ends of fingers and toes of a bluish black color; the nails are clubbed and curved; veins on chest prominent; temperature normal; pulse 104, small but equal in both wrists. The heart dullness extends one inch to the right of the sternum, and the apex beat is seen, felt and heard most distinctly in the sixth intercostal space, about two inches to the right of the nipple and one-half inch to the left of the sternum. No murmur is heard at the apex, but a systolic murmur is heard clearly over the base of the heart, loudest in the second and third intercostal spaces, apparently equal in intensity on both sides of the sternum and faintly heard in the neck and near the lower angle of the left scapula. Both the aortic and pulmonary second sounds seem exaggerated. The examination of the other organs gave a negative result, except that the liver is somewhat enlarged. Shortly after this examination, an opportunity came to witness a mild example of the attacks already alluded to. While walking slowly on the street he complained of feeling tired, his face became dark blue and his lips and finger tips almost black, the heart beat violently, but irregularly, and he would have fallen unless supported. The breathing was panting. On being taken to a convenient place and allowed to lie down, he became very drowsy and lay quietly for some minutes, the blueness gradually lessening. Pain was slight in this attack. The mother states that shortly after, on again attempting to walk, the attack was repeated in a severer form.

Since writing the above, the patient has had one attack of convulsions, which occurred about the 1st of June. For a few days previous, he had been more quiet and had been more troubled with dyspnoea and cyanosis. The convulsions were general, and he was unconscious for some minutes. When seen a few hours later, he seemed perfectly natural. There has been no return of the convulsions as yet.

Cases of congenital heart lesion are of sufficient rarity to warrant more than a passing study. But although the diagnosis, as in this patient, may be easy, the exact lesion existing in a given case is generally difficult and often impossible to determine. The condition is due to arrested development or to endocarditis in foetal life or to both combined. The arrest in development may take place at any stage in the growth of the heart. If, however, the heart remains in a very rudimentary state, the child can survive only a few hours or, at the most, a few days. If the arrest in development occur at a later period, as is generally the case, the normal mechanism of the heart and large blood vessels may be more nearly complete. The cardiac partitions may be deficient, or the large vessels misplaced or even transposed. Thus the aorta may be displaced to the right arising in part or wholly from the right ventricle, while the pulmonary artery may arise from the left ventricle. Eustace Smith, in his "Diseases of Children," says, "When the aorta is merely displaced to the right, without malposition of the pulmonary artery, we usually find some obstruction to the passage of blood from the right ventricle through the latter. The artery is too small, or its valves are incomplete, or the blood is prevented from passing freely into it by some constriction of the ventricle near its outlet, or its channel may be entirely obliterated, in all of which cases the foramen ovale must remain open, or the circulation could no longer be carried on. The blood, being unable to find its way in sufficient quantities to the left side of the heart through the lungs, continues to follow its original course through the opening in the auricular septum, and the foramen ovale remains open. If, however, the aorta arises sufficiently to the right to allow the escape of blood through it from the right ventricle, the foramen ovale and ductus arteriosus may cease to be pervious.

"Constriction in the pulmonary artery, with deficiency in the septum of the ventricles, so that the aorta communicates with the right ventricular cavity, is the commonest form of congenital malformation of the heart. Sometimes the descending aorta arises from the pulmonary artery, being apparently a continuation of the ductus arteriosus. In this case, a small ascending aorta arises from the left ventricle to supply the head and neck by the usual vessels."

On the other hand, the defect may consist, not in the foetal openings remaining patent after birth, but in their closing too early and before birth. Should such be the case with the foramen ovale, all the blood must pass through the pulmonary artery and the ductus arteriosus. Hence, the right heart becomes immensely hypertrophied, the left side being unnaturally small. Or if the ductus arteriosus closes too early, the aorta may arise from the right ventricle and give branches to the lungs, the pulmonary artery remaining rudimentary. The congenital disease may also consist in narrowing of the openings of the large vessels or in defective valves, either from malformation or from foetal endocarditis, which almost always attacks the right heart, this being more active than the left in foetal life. In all cases, the heart, and especially the right side, is greatly hypertrophied. In attempting to diagnosticate the exact condition which exists in the heart in these cases, we are at once placed under a formidable disadvantage in that, instead of having, as usual, a heart of which we know the structure, the number and situation of its openings and the number and working of its valves, and instead of having a normally directed blood current, in which conditions the physical signs have, consequently, a definite meaning, we are dealing with an organ in which the number and position of the openings and the direction of the blood current is unknown except by conjecture. Nevertheless, certain rules must be born in mind. In the first place, some forms of congenital defect are quickly fatal. An infant whose heart consists of only two cavities will probably not live a month. Transposition of the aorta and pulmonary artery also brings death within two or three years, and generally within one year. The same is true where the aorta arises from the pulmonary artery. If the child has survived three years, the above conditions can be excluded. Some contraction at the orifice of the pulmonary artery is, then, the most common lesion, probably associated with patency of the foramen ovale and, perhaps, with a connection between the aorta and right ventricle and patency of the ductus arteriosus. The physical signs of this condition are a systolic murmur heard over the base of the heart, especially over the pulmonary valves, not transmitted into the large vessels, associated with enlargement of the right side. The fact that, in the case reported, the murmur is apparently transmitted to the large vessels seems to indicate that some of the above may not be the only pathological conditions present.

An open foramen ovale is seldom a solitary abnormality, although congenital cyanosis without a murmur, or with a very faint one, and no enlargement of the right heart in a child at least three years old, points to such a condition. Hochsinger says: "The entire absence of murmurs at the apex, with their evident presence in the region of the auricles and over the pulmonary orifice, is always an important element in differential diagnosis and points rather to septum defect or pulmonary stenosis than to endocarditis." Osler says that stenosis, obliteration and atresia at the pulmonary orifice, or stenosis of that portion of the right ventricle adjoining it, with or without imperfection of the ventricular septum, patency of the foramen ovale and persistence of the ductus arteriosus, constitute the most important group of all congenital heart affections, and 119 of the 181 cases of congenital heart anomalies collected by Peacock came under this class, as did also eighty-six per cent. of those cases living beyond the twelfth year. DaCosta makes a similar statement.

But all authors seem to agree that an exact diagnosis can not be made, and that other conditions than those diagnosticated are liable to be present.

As regards symptoms, the more common ones have been well represented in this case. The most striking one is the livid skin or cyanosis. This may not be present at birth, especially to any considerable extent. The extremities of the fingers and toes generally show it, however, although it may be weeks or months before it becomes sufficiently general to excite attention. What causes the cyanotic appearance? Hunter attributed it to intense mixing of the arterial with the unoxygenized blood. This view has been shown to be incorrect. In the first place, cyanosis may occur where there is no mixing of arterial and venous blood, and in the second place the degree of cyanosis is not dependent on the amount of venous blood contaminating the arterial current. Morgagni and Peacock were the first to show the true cause to be intense general congestion, due to stasis of blood in capillaries dilated by long standing congestion, aided by imperfect aeration of the whole mass of blood.

What is the prognosis of these cases? It depends largely, of couse, on the degree of obstruction to the circulation, but it is generally very unfavorable. One-half of these cases die in the first year and only about one-third survive the second year. On account of the constant and intense venous congestion, the functions of all the organs are imperfectly performed. The child is poorly nourished, thin in flesh, and constantly liable to catarrhal troubles of the respiratory and digestive tract. The establishment of respiration at birth, the eruption of the teeth, weaning, and the diseases peculiar to childhood are always, at best, trying periods, while in a child with congenital heart disease they are periods of great danger. Occasionally, however, these cases live to adult life. Cyanotic children often die in convulsions or from heart failure in the attacks of syncope. Pneumonia and tuberculosis are rather frequent complications, and DaCosta calls attention to abscess of the brain occurring in cases of malformed heart without apparent cause. Certain symptoms are hence especially unfavorable, such as frequent attacks of syncope, great drowsiness, convulsions, or other signs of brain irritation, as well as symptoms of renal disease.

Regarding treatment, it is evidently important to keep the unfortunate patient, to as great an extent as possible, from being overtaken by the diseases and difficulties to which he is so peculiarly liable. On account of the lack of vitality, the cold extremities, and the danger of contracting cold, warm clothing is necessary as well as protection from changes of temperature and inclement weather, while at the same time the advantage of fresh air, in keeping up the general health, is to be borne in mind. Again, on account of the malnutrition, care as to diet is essential, that the patient may, on the one hand, be as well nourished as possible, and, on the other, that he may be guarded from the ills arising from his natural tendency to gastro-intestinal catarrh. Hence, an occasional mercurial, to relieve, to some extent, the overloaded portal system, is judicious. Freedom from excitement and over-exertion, as well as speedy and careful attention to all ailments, however slight, are important points. Owing to the natural dryness of the skin, a bath should be given at least once daily, followed by brisk friction. For violent attacks of palpitation, digitalis may be guardedly tried, and for the attacks of syncope and dyspnoea stimulants and ammonia are indicated.

The urine should be watched in order that the appearance of albumen, or other evidence of renal disturbance, may suggest prompt treatment.

---

LIGATURE OF THE SPERMATIC CORD IN THE TREATMENT OF HYPERTROPHY OF THE PROSTRATE GLAND.—In a paper read before the Philadelphia Academy of Surgery in November, 1894, Ewing Mears held that to obliterate the function of the generative apparatus would be a rational method of treatment in ordinary forms of prostatic hypertrophy. Without doubt, he stated, castration would prove effectual in the production of atrophy; but to this operation patients would naturally refuse to submit unless in advanced stages of bladder disease resulting from prostatic obstruction. Ligature of the vas deferens was suggested as an operation which would probably be as efficacious as castration and be more readily acceptable. The author has seen the report of one case in which this operation had been performed with a successful result. The gradual disappearance of the sexual function, the author pointed out, would not be so liable to disturb the mental condition of the patient if the testes were preserved. In every case the patient should be informed of the character of the operation and what is intended to be accomplished by it. The author regards it as the duty of the surgeon to urge very earnestly the performance of any operation which will be efficacious in terminating the horrible sufferings of those suffering from the results of prostatic obstruction.—*British Medical Journal*.

